# (Predictable) performance bias in unsupervised anomaly detection

**DOI:** 10.1016/j.ebiom.2024.105002

**Published:** 2024-02-09

**Authors:** Felix Meissen, Svenja Breuer, Moritz Knolle, Alena Buyx, Ruth Müller, Georgios Kaissis, Benedikt Wiestler, Daniel Rückert

**Affiliations:** a Chair for AI in Healthcare and Medicine, Klinikum rechts der Isar der Technischen Universität München, Einsteinstr. 25, Munich, 81675, Germany; b Department of Science, Technology and Society, School of Social Sciences and Technology, and Technical University of Munich, Arcisstr. 21, Munich, 80333, Germany; c Department of Economics and Policy, School of Management, Technical University of Munich, Arcisstraße 21, 80333, Munich, Germany; d Konrad Zuse School of Excellence in Reliable AI, Munich Data Science Institute (MDSI), Walther-von-Dyck-Str. 10, Garching, 85748, Germany; e Institute for History and Ethics of Medicine, School of Medicine, Technical University of Munich, Prinzregentenstraße 68, Munich, 81675, Germany; f Institute for Machine Learning in Biomedical Imaging, Helmholtz Munich, Ingolstädter Landstraße 1, 85764, Neuherberg, Germany; g Department of Diagnostic and Interventional Neuroradiology, Klinikum rechts der Isar, Ismaninger Str. 22, Munich, 81675, Germany; h Department of Computing, Imperial College London, London, SW7 2AZ, UK; i TranslaTUM, Center for Translational Cancer Research, Technical University of Munich, Ismaninger Str. 22, Munich, 81675, Germany

**Keywords:** Artificial intelligence, Machine learning, Algorithmic bias, Subgroup disparities, Anomaly detection

## Abstract

**Background:**

With the ever-increasing amount of medical imaging data, the demand for algorithms to assist clinicians has amplified. Unsupervised anomaly detection (UAD) models promise to aid in the crucial first step of disease detection. While previous studies have thoroughly explored fairness in supervised models in healthcare, for UAD, this has so far been unexplored.

**Methods:**

In this study, we evaluated how dataset composition regarding subgroups manifests in disparate performance of UAD models along multiple protected variables on three large-scale publicly available chest X-ray datasets. Our experiments were validated using two state-of-the-art UAD models for medical images. Finally, we introduced subgroup-AUROC (sAUROC), which aids in quantifying fairness in machine learning.

**Findings:**

Our experiments revealed empirical “fairness laws” (similar to “scaling laws” for Transformers) for training-dataset composition: Linear relationships between anomaly detection performance within a subpopulation and its representation in the training data. Our study further revealed performance disparities, even in the case of balanced training data, and compound effects that exacerbate the drop in performance for subjects associated with multiple adversely affected groups.

**Interpretation:**

Our study quantified the disparate performance of UAD models against certain demographic subgroups. Importantly, we showed that this unfairness cannot be mitigated by balanced representation alone. Instead, the representation of some subgroups seems harder to learn by UAD models than that of others. The empirical “fairness laws” discovered in our study make disparate performance in UAD models easier to estimate and aid in determining the most desirable dataset composition.

**Funding:**

10.13039/501100000781European Research Council Deep4MI.

**Research in context**

**Evidence before this study**

We searched PubMed, Scopus and Google Scholar for Machine Learning and Deep Learning studies related to “unsupervised anomaly detection”, “fairness”, “model bias”, and “intersectionality” before August 2023. The list of publications was complemented by the authors’ knowledge of the body of literature and suggestions from colleagues. Several of these prior works have investigated the fairness of supervised classification models regarding protected attributes, such as gender, age, or race, and how this negatively affects patient care. Dataset composition, fairness in population-wide studies, and the effect of intersectionality (considering multiple protected attributes) have been thoroughly investigated for this class of models. These studies have found lower performances in under-represented or socio-economically disadvantaged patient groups, as well as disproportionate impacts in intersectional subgroups (i.e., individuals who share more than one sensitive trait).

**Added value of this study**

Unsupervised anomaly detection (UAD) differs from supervised classification not only algorithmically but also regarding the context in which it is applied: the main application of UAD is pre-screening or triaging to support clinicians in handling increasing amounts of imaging data, and therefore has a distinct role. Since UAD models learn the training data-generating distribution instead of an association between input and output labels, the effects for under-represented subpopulations are likely to be different from supervised models. To this end, our work investigates and quantifies fairness in UAD models. Our experiments reveal empirical “fairness laws”—linear relationships between dataset composition and subgroup performance that facilitate the *a priori* estimation of subgroup performance. The presented empirical results further show severe performance differences for subgroups even under balanced training data, suggesting that performance disparities in UAD can not be eliminated by equal data representation alone. Lastly, we introduce subgroup-AUROC (sAUROC) to better quantify performance discrepancies for subgroups in machine learning models.

**Implications of all the available evidence**

This study shows that performance bias is not limited to supervised classification models and further suggests additional care and rigour will be necessary in designing and deploying UAD algorithms to minimise excessive risk for misdiagnosis of different subpopulations. This also implies that new UAD models generally need to be evaluated regarding their fairness. We have further shown that performance bias behaves predictably in UAD. The above-mentioned “fairness laws” render subgroup performance in UAD more predictable and can help to guide data collection towards more fair models.

## Introduction

In unsupervised anomaly detection (UAD), a machine learning (ML) model is trained to capture a distribution of training samples with the aim of being able to identify outliers/anomalies that do not stem from the distribution underlying the training data-generating process. Within the medical domain, UAD serves as a valuable tool for detecting pathological samples while only requiring data derived from healthy patients without any disease-specific labels.^1^, ^2^, ^3^ This approach allows for making use of the vast amounts of clinically unremarkable data acquired in hospitals on a daily basis. In contrast to supervised methods, UAD, by construction, avoids the problem of class/label imbalance, making it well-suited to identify even rare anomalies, for which the collection of sufficient training data would otherwise present a challenge. A UAD model Φ produces an anomaly score a for a data point x. Formally: Φ(x)=a. The model assigns higher anomaly scores to samples far from its learned distribution.

Recently, a lack of dataset diversity defined through, e.g., age, sex, or ethnicity, has been recognised as an important concern in medical imaging^4^ and beyond,^5^ as ML models trained on such data often provide biased predictions that result in poor performance on under-represented subgroups. Furthermore, there is unequivocal evidence that this bias is harmful to patients: In a diverse dermatology dataset, Dansehjou et al. found that most state-of-the-art ML models used for skin cancer detection exhibited significantly lower performance than previously reported, particularly on dark skin tones. In a clinical deployment setting, this can lead to delayed or missed diagnoses for patients with darker skin, potentially resulting in inadequate treatment and poorer outcomes.^6^ This issue becomes even more pronounced when taking intersectionality into account: Both Seyyed-Kalantari et al. and Stanley and colleagues independently reported that performance disproportionately worsens for patients belonging to multiple subgroups already adversely affected.^7^^,^^8^

**Fig. 1:**
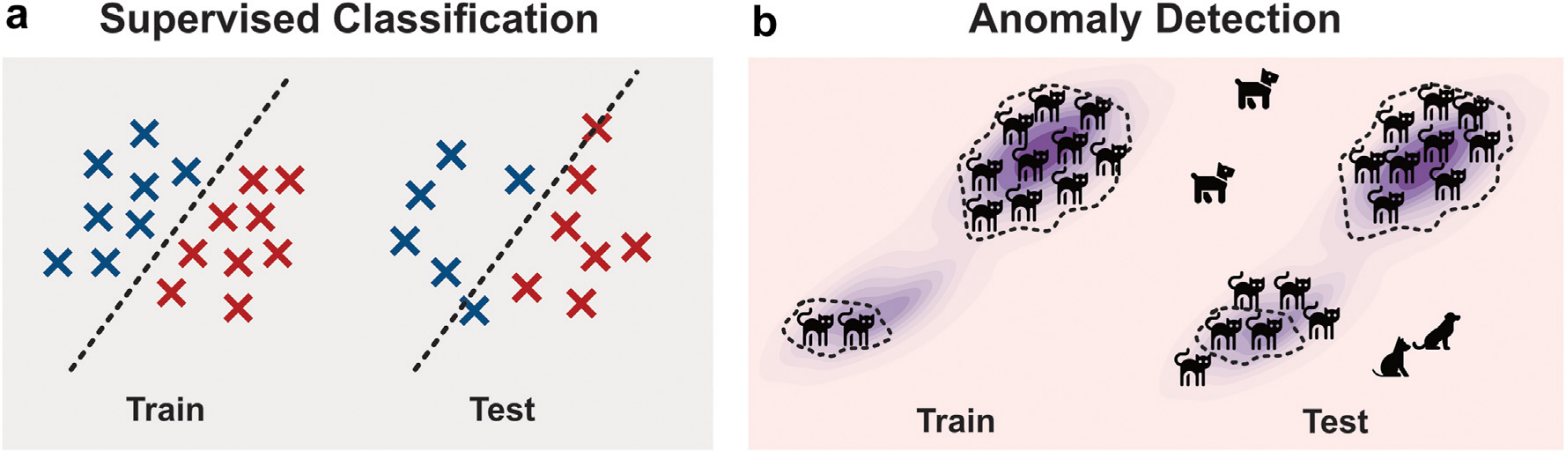
Supervised classification models (left) learn a mapping between input (samples) and (disease) labels and thus require annotated data. On the other hand, UAD models (right) learn to capture the distribution of training samples (healthy), here visualised as cats for exemplary purposes. If a subgroup is not adequately represented in the training data, the UAD model will assign data samples from that group higher anomaly scores, resulting in more false positives at test time.

For UAD models, which are trained to learn a “normal” distribution against which to contrast “anomalies” (see Fig. 1), imbalanced training data is particularly challenging. This is because subgroups observed less frequently in the training data lack sufficient representation for the UAD model to accurately learn their normal patterns, resulting in higher anomaly scores and, consequently, higher false-positive rates. These can be detrimental in numerous ways, from unnecessary diagnostic tests and interventions to the potential for misdiagnosis or delayed diagnosis of true findings. However, while the fairness of supervised ML models for clinical application has been increasingly studied,^4^^,^^7^^,^^9^ and the problem has been occasionally discussed in the UAD literature,^10^^,^^11^ a thorough empirical investigation of the matter for UAD models does not yet exist.

The main contribution of our study is a thorough investigation into the fairness of UAD models by measuring how the proportion of a demographic subgroup in the training data of UAD models affects the resulting performance. We operationalise our findings and introduce empirical “fairness laws”^k^ (i.e., a linear relationship between subgroup representation and performance), which help to identify the optimal dataset compositions for training fairer UAD models. Finally, we introduced subgroup-AUROC (sAUROC) as a way to better evaluate fairness in ML models.

## Methods

### Datasets

We utilised three large public chest X-ray datasets to measure the fairness of UAD models regarding the protected attributes sex, age, and race: MIMIC-CXR-JPG (MIMIC-CXR),^13^ ChestX-ray14 (CXR14),^14^ and CheXpert.^15^ MIMIC-CXR contains 371,110 chest X-rays from a cohort of 65,079 patients acquired at Beth Israel Deaconess Medical Centre in Boston, Massachusetts, USA, between 2011 and 2016. The CXR14 dataset, collected from the NIH Clinical Centre in Bethesda, Maryland, USA, between 1992 and 2015, includes 112,120 frontal-view chest radiographs from 30,805 distinct patients. The CheXpert database contains 224,316 chest radiographs of 65,240 patients acquired at Stanford Hospital between October 2002 and July 2017.

All three datasets contain structured diagnostic labels (12 labels for MIMIC-CXR and CheXpert, 13 for CXR14, excluding the “support devices” label) and a “no finding” or “normal” label marking the absence of any other identified diagnostic labels. These labels were automatically derived from the associated radiology reports using natural language processing techniques. In addition, demographic metadata about the patients’ age (MIMIC-CXR: 60 ± 18, CXR14: 47 ± 17, CheXpert: 60 ± 18 years) and their gender (MIMIC-CXR: 47.7%, CXR14: 43.5%, CheXpert: 40.6% images of female and 59.4% of male patients^l^) was available. While the term *gender* was used in the datasets, given the nature of the X-ray images revealing anatomical traits inherent to biological sex, we will use the term *sex* as a more precise descriptor in this context. For MIMIC-CXR, additionally, the self-reported race was available from Johnson et al.^16^ Here, 17.3% of the patients identified as “Black” and 62.8% as “White”. While information about ethnicity was also available for CheXpert, the dataset sizes for the experiments described in Section 2 were insufficient to adequately train anomaly detection models (∼500 images for training).

### Dataset construction and inclusion criteria

To ensure consistency in the inclusion criteria, distinct selection strategies were employed for all datasets. We only considered frontal-view images without support devices and further excluded those with all labels marked as uncertain. Detailed dataset demographics can be found in the Supplementary Material in Table S1. All images were centre-cropped and resized to 128 × 128 pixels.

**Fig. 2:**
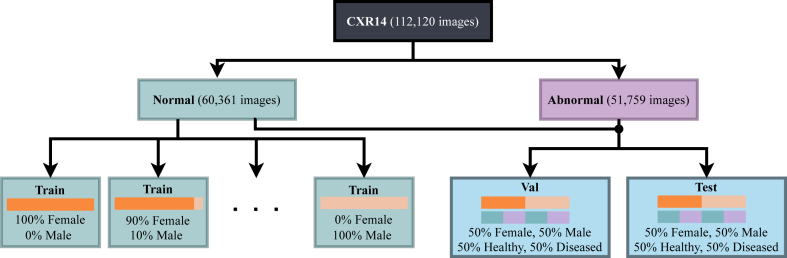
Exemplary flowchart of the procedure to generate training, validation, and test sets for CXR14.

We constructed the training datasets using only the “no finding” and “normal” labels. All other diagnostic labels were consolidated into a “diseased” label. First, validation and test sets were randomly generated with equal representation of normal and abnormal classes and equal distribution of the protected attributes. From the remaining normal data, we created training sets with varying proportions of the protected attribute subgroups (from 0% to 100%). In this process, the total number of training samples was held constant (e.g., the training set with 50% *male* and 50% *female* samples contains the same number of samples as the one with 10% *males* and 90% *females*). The dataset construction for CXR14 is illustrated in Fig. 2. For MIMIC-CXR and CheXpert, the procedure was analogous. Importantly, there was no patient overlap between the training, validation, and test sets. The numbers of samples in the training, validation, and test sets of all considered dataset and protected attributes can be found in the Supplementary Material in Table S2.

We selected two variables for the protected attribute race: *white* and *black*. The group *white* consists of the variable “WHITE”. We accumulated four variations of self-reported race into the group *black*: “BLACK/AFRICAN AMERICAN”, “BLACK/CAPE VERDEAN”, “BLACK/AFRICAN”, and “BLACK/CARIBBEAN ISLAND”. Further relevant subgroups, such as Asian or Latin, were excluded due to the small number of available images. The categorisation along the sex dimension was taken from the binary *male* and *female* values provided in the datasets. For the separation between *young* and *old* patients, we opted for a data-driven approach, dividing the patient pool into three age groups based on the maximum age within the MIMIC-CXR dataset and removing the centre group to ensure a sufficient age gap between the patients in the two remaining groups. This stratification resulted in individuals up to 31 years being classified as *young*, while individuals aged 61 years and above were categorised as *old* within our analysis.

We conducted additional experiments to examine fairness in intersectional groups, controlling for multiple protected attributes (sex, age, and race) in the MIMIC-CXR dataset. We constructed random test sets for each possible combination of two protected attributes (i.e., *male* and *white*, *male* and *black*, *female* and *white*, etc.), each balanced in terms of both positive and negative samples, and all considered protected attributes. The remaining normal data was used to form the training set. While this training set was unbalanced regarding the protected attributes, it approximates the population of the originating hospital. Details about the dataset sizes for this experiment are in the Supplementary Material in Table S3.

Disease prevalence is unequal in the subgroups defined above. This prevalence shift is known to cause disparities in many metrics, such as the receiver operating characteristics (ROC) curve.^17^ Since we intend to measure model performance bias in this study, we corrected for unequal prevalence while constructing our validation and test sets. Since the prevalence in the datasets considered suffer from selection bias and, thus, likely do not represent the true prevalence in many real-world scenarios, we chose an arbitrary but consistent prevalence of 0.5 in the test sets.

### Statistics

Since samples of under-represented subgroups are likely to yield higher anomaly scores and, consequently, are more often falsely flagged as positive (c.f. Section 1), we considered predictive equality (or false positive error rate balance) as the most relevant measure to assess (group-) fairness in the context of UAD.

While the effect of generally higher or lower anomaly scores for a subgroup could potentially be partially mitigated *post hoc* by selecting a unique threshold for each group, this solution can not be used in many cases where the association of a sample to a particular variation is unavailable, for example in retrospective cases. The amount of required thresholds further grows combinatorically with the number of protected variables in intersectional subgroups, while the number of available samples to estimate this threshold shrinks simultaneously.^7^ Thus, when evaluating an anomaly detection model's fairness regarding multiple subgroups, metrics such as the false positive rate at a minimally required true positive rate (FPR@x%TPR) cannot be calculated separately for each group. Instead, the threshold necessary to achieve the minimum TPR must be computed across the entire dataset, while the resulting FPRs should be determined individually for each subgroup.^17^ Similarly, the area under the receiver operating characteristics curve (AUROC) can not be computed for each group separately, as the minimum and maximum anomaly scores for both groups are likely significantly apart. To alleviate this issue, we propose the subgroup-AUROC (sAUROC), which can be viewed as a threshold-free extension of the FPR@x%TPR metric.

For a subgroup s∈S in the overall population P, the sAUROC is calculated as follows: For every decision threshold *t*, the TPR is computed over the whole population, but the FPR only over the specific subgroup. Mathematically, sAUROC can be described as:

$${\text{TPR}}_{P}\left(t\right)=\frac{{TP}_{P}\left(t\right)}{{TP}_{P}\left(t\right)\;+\;{FN}_{P}\left(t\right)}$$

$${\text{FPR}}_{P}\left(t\right)=\frac{{FP}_{P}\left(t\right)}{{FP}_{P}\left(t\right)\;+\;{TN}_{P}\left(t\right)}\text{,}$$

where TP(t), FN(t), FP(t), and TN(t) are the numbers of true positives, false negatives, false positives, and true negatives at threshold *t*, respectively, and the subscripts P and *s* denote if all samples are considered, or only the ones from the subgroup *s*, respectively. Finally, the sAUROC is computed as

$$sAUROC\left(s\right)=\int_{0}^{1}{TPR}_{P}\left({FPR}_{s}^{-1}\left(x\right)\right)dx\;\text{.}$$

sAUROC paints a thorough picture of performance differences between subgroups of a population. We ran each experiment ten times with different random seeds.

### Anomaly detection model

In our experiments, we employed the Structural Feature Autoencoder (FAE) by Meissen et al.,^18^ the best performing method among contemporary state-of-the-art techniques in a recent comparative analysis by Lagogiannis et al.^3^ Model optimisation was performed using the Adam optimiser, with a learning rate of 0.0002, for 10,000 iterations. Each experiment was run with ten different random seeds to compute confidence intervals using a Gaussian-based approximation and to perform statistical significance tests. To validate that our findings are not specific to the chosen model, we validated our results with the Reverse Distillation model (RD) by Deng et al.^19^—the second-best model in Lagogiannis et al.^3^ The settings for both models were preserved at their default parameters. These can be found in the Supplementary Material in Tables S4 and S5. For details regarding the two models, we refer the reader to the original works.

### Ethics

This research is exempt from ethical approval as the analysis is based on secondary data which is publicly available, and no permission is required to access the data.

### Role of the funding source

The funders had no role in study design, data collection, data analysis, data interpretation, or writing of the report. The authors had full access to all the data in the study and had final responsibility for the decision to submit for publication.

## Results

### Subgroup representation and performance are linearly correlated

**Fig. 3:**
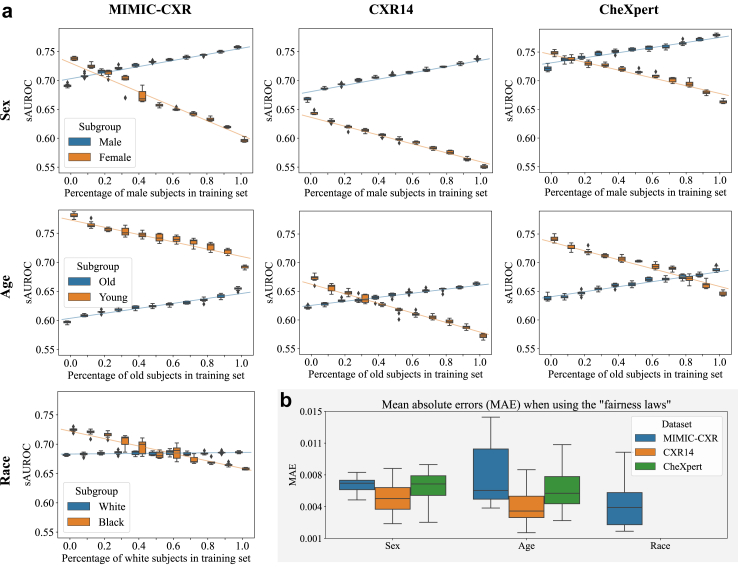
a) A linear relationship between the representation of a subgroup in the training dataset and its performance was observed across all datasets and subgroups. Equal representation of subgroups did not produce the most group-fair results. Experimental results for the FAE on the MIMIC-CXR, CXR14, and CheXpert datasets trained under different sex, age, or race imbalance ratios. Each box extends from the lower to upper quartile values of ten runs with different random seeds with a line at the median. Regression lines along the different imbalance ratios are additionally plotted. The exact numbers can be found in the Supplementary Appendix. b) The mean absolute errors (MAE) between the real subgroup performances and those estimated using the “fairness laws” for each dataset and protected variable. Each box again shows the results over ten runs with different random seeds.

Fig. 3 shows a significant correlation between the representation of a subgroup in the training dataset and the subsequent performance for that subgroup (Pearson correlation coefficients of 0.979 ± 0.011, 0.971 ± 0.016, and 0.682 ± 0.331 in the sex, age, and race experiments respectively). This relationship was linear in all experiments, which allowed us to accurately estimate the sAUROC-performance for training with any composition of patients regarding a protected attribute, using linear interpolation from the extreme values (0% and 100%)—with a mean absolute error of 0.0061 ± 0.0029 for age, 0.0063 ± 0.0015 for sex, and 0.0045 ± 0.0025 for race. Only in the race-controlled experiments on MIMIC-CXR, the influence of the dataset composition on the performance outcome for patients from the *white* group was much weaker (Pearson correlation coefficient 0.398 ± 0.241). The detailed results of all experiments and the correlation coefficients per subgroup and class are provided in the Supplementary Material in Tables S6–S8 and Table S9, respectively. The results of the RD model in the Supplementary Material, Fig. S1 and Tables S10–S13, showed the same linear behaviour.

While the overall anomaly detection performance varies between datasets, the above-described effect is consistent for each demographic subgroup. The slopes of the regression lines are 0.050 ± 0.004 for *male*, −0.089 ± 0.024 for *female*, 0.040 ± 0.003 for *old*, and −0.074 ± 0.008 for *young*, respectively. Notably, the slopes for *female*, *young*, and *black* are steeper than for *male*, *old*, and *white*, respectively.

### Unfairness exists with balanced training data

The experiments in Fig. 3 also revealed that *male* subjects consistently received significantly higher scores across all datasets, even under balanced conditions, where both subgroups are equally represented in the training data (Welch's t-test, *N* = 10 different runs, *p <* 0.01). This pattern also held true for *old* patients, except for the MIMIC-CXR dataset, where *young* individuals obtained significantly higher scores (Welch's t-test, *N* = 10 different runs, *p <* 0.01). Only when the protected variable was the patients' self-reported race, balanced training data did not lead to significant unequal performance in the FAE model (Welch's t-test, *N* = 10 different runs, *p* ≥ 0.01).

### Unfairness is amplified in intersectional subgroups

**Fig. 4:**
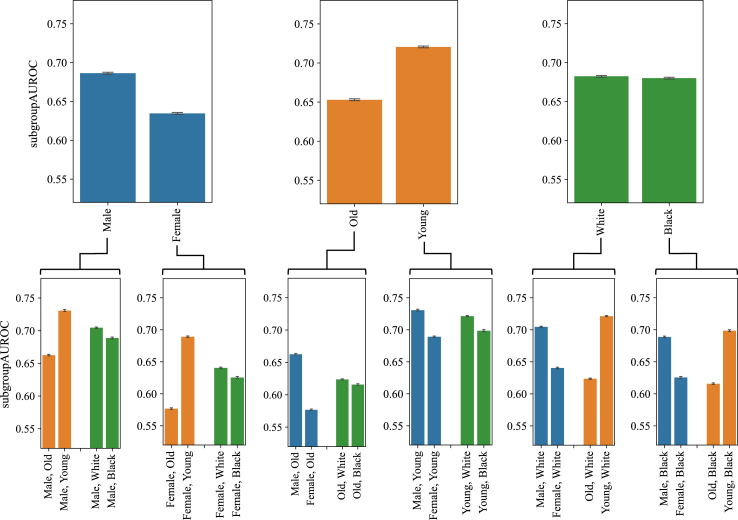
In the MIMIC-CXR dataset, representative of the Beth Israel Deaconess Medical Center, Boston, USA, diseases were detected better in *male* than *female* patients and in *young* than *old* patients. When considering a second demographic variable, these differences were amplified, e.g., the difference between *male* and *female* subjects is larger among older patients than younger ones. Top row: *male* vs. *female*, *old* vs. *young*, and *white* vs. *black*. Bottom row: intersectional subgroups. Each bar shows the mean and standard deviation over ten runs with different random seeds.

Our intersectional experiments’ outcomes are illustrated in Fig. 4. Given that the training dataset was not controlled for any protected variable, the findings presented here revealed potential unfairness within a population representative of the Beth Israel Deaconess Medical Centre. Here, *male* patients received higher scores than *female* patients, and *young* patients achieved higher scores than their *old* counterparts. The performance of *white* and *black* patients appears to be equivalent. This pattern is also consistently observable in the intersectional subgroups featured in the lower row of the figure, although *black* patients.

Scored slightly lower in these cases. Moreover, the score disparity (*Δ*) between *male* and *female* patients was more pronounced among *old* individuals compared to their *young* counterparts (*Δ*0.085 and *Δ*0.042, respectively). Similarly, the score disparity between *old* and *young* patients was larger among *female* patients in comparison to *male* patients (*Δ*0.112 and *Δ*0.068, respectively).

### Naive AUROC fails to capture “fairness laws”

**Fig. 5:**
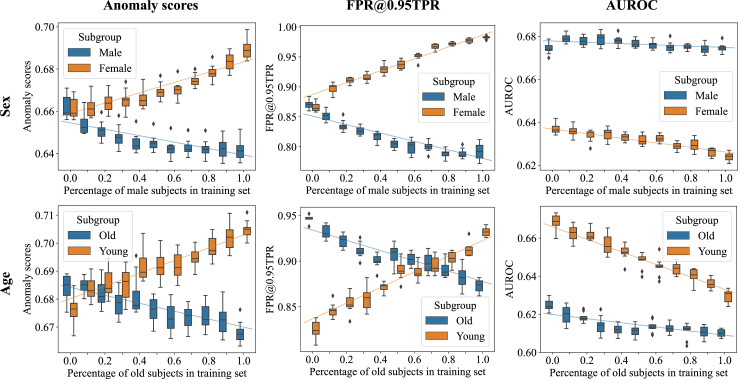
The representation of a subgroup in the training dataset had a strong influence on its anomaly scores, the false positive rate at a minimally required true positive rate, and our proposed sAUROC (c.f. Fig. 3). The naive computation of AUROC did not capture this relationship. Anomaly scores (left), FPR@0.95TPR (middle), and naive AUROC (right) for different compositions of sex (top) and age (bottom) on the CXR14 dataset.

To put the sAUROC results in perspective, we additionally show the anomaly scores, FPR@0.95TPR, and naive AUROC for different dataset compositions on CXR14 in Fig. 5. The anomaly scores for a subgroup increased as their representation in the training data shrank. FPR@0.95TPR exhibited analogous behaviour. For AUROC, an increase in samples from one subpopulation did not improve scores for that group. Instead, an increase in *male* or *old* patients resulted in similar or worse scores for all groups.

## Discussion

It has been shown that an embedded ethics and social science approach is helpful when analysing complex, interdisciplinary problems like the one discussed in this work.^20^^,^^21^ We, therefore, drew on the interdisciplinary expertise of the authors in the discussion of the results, combining technical, social, and medical perspectives.

The experiments in Section 3 have unveiled “fairness laws” for UAD models: linear relationships between the representation of a subpopulation in the training data and the performance of that group (c.f. Fig. 3). These relationships enable practitioners to accurately predict a subgroup's performance using only two points of measurement and linear inter- or extrapolation. This implies that the optimal dataset combination under any fairness constraints can be easily estimated beforehand based on the above described linear relationship.

However, like supervised methods,^8^ UAD models suffered from compounding adverse effects in intersectional subgroups (c.f. Fig. 4). For example, the difference we found in model performance between *male* and *female* patients was larger in *old* patients than in *young* ones, resulting in *old female* patients being at a particular disadvantage.

Our experiments demonstrated substantial performance disparities among subgroups, even when they were equally represented in the training data. For instance, *male* patients on CXR14 consistently received significantly higher scores than their *female* counterparts (*male*: 0.71, *female*: 0.60), and *young* patients outperformed *old* ones on MIMIC-CXR (*young*: 0.73, *old*: 0.63). Notably, the dataset compositions that yield the most group-fair results—as measured by predictive equality—were often situated towards the extremes of the dataset composition spectra. In CheXpert and MIMIC-CXR, optimal sAUROC parity was achieved with a 70% and 80% *female* patient representation, respectively, whereas for CXR14, the composition that led to the most group-fair outcome did not include any *male* samples. This leads us to the hypothesis that there might be subgroup-specific differences that cause some subgroups to be easier to represent by the UAD model than others, as discussed by Nalisnick et al.^22^ Further potential reasons for this performance gap are summarised in a recent review by Petersen et al.^23^ Among them are systematic labelling errors, as well as potential inherent task difficulty differences between groups. Our findings, therefore, highlight the need for medical expertise in evaluating these models and their potential performance biases.

Disease detection is the central first step in the diagnostic process.^24^ Coupled with the increasing demand for medical imaging, UAD models fill a relevant clinical need. In this pivotal role, unfairness in UAD, perhaps even more so than “downstream”, more specialised models, has significant potential to negatively affect patients. Our experiments revealed that UAD models produce elevated false-positive rates for some subgroups (c.f. Fig. 5). False positives can cause serious harm to patients, such as unnecessary follow-up tests (and costs),^25^ harm from unnecessary treatment, and psychological distress,^26^ and can generally cause distrust in the model or ML techniques in general.

The results in Fig. 5 show why sAUROC is a more suitable measure to assess the fairness of ML models. The figure displays a linear relationship between anomaly scores and dataset composition, indicating that, as groups get less represented in the training data, they are flagged as “more anomalous” by the model. These relationships were also reflected in the FPR@0.95TPR and sAUROC. The naive, individual calculation of AUROC for each subpopulation, however, did not exhibit this expected pattern. While the anomaly scores clearly showed disparities but were missing information about the resulting classification performance differences and FPR@0.95TPR is only a point measure considering only a single decision threshold, sAUROC painted a more comprehensive picture by considering all possible thresholds while capturing disparate performances.

The findings of our work need to be viewed with an awareness that the categories of human differences we are working with are complex and historically formed. We could not find comprehensive information about how subjects were assigned labels of sex and race in the datasets. This leaves us unclear about what social and biological aspects were included in these categories, important information for nuanced analyses that take into account how human bodies are shaped by both social and biological factors.^27^^,^^28^ Further, the labels of the datasets used for evaluation in this study are at risk of being biased. Reasons for that reach from biases of medical professionals in the creation of the radiology reports^29^ to the automatic label extraction from these reports using a rule-based natural language processing system, which is known to generally contain high levels of label noise, especially in the oldest patient group.^30^ Although UAD models only require minimal labels during training (healthy vs. diseased) and thus are presumably less susceptible to systematic labelling errors during training than supervised models, such errors might have an effect on our experimental results when they occur in the evaluation data. Due to the small sample sizes of many ethnic subgroups in the available databases, our analysis of the race dimension was restricted to the categories *white* and *black* to guarantee meaningful insights of our results. While we assume analogous effects on other racial categories like *asian*, *latin*, etc., the shortage of available data prevented us from empirically substantiating this hypothesis. This limitation reflects the bias against under-represented racial groups that exists in current public medical data sets. In conclusion, this study represents an effort to quantify fairness in UAD on a large scale, including the results of 1560 trained models. Our extensive experiments on various large-scale datasets and protected attributes confirmed that a demographic subpopulation's anomaly detection performance strongly depends on its representation in the training data and can be efficiently estimated, simplifying the task of identifying the fairest composition. Our experiments further showed that disparate performance between two subgroups can not solely be explained by the under-representation of one subgroup. Instead, some subgroups seemed to be harder to learn by the UAD models than others and, thus, were generally flagged as “more anomalous”. While this study has found performance disparities existing along the three considered variables, likely, more of them exist (for example, people with disabilities). Thus, enhancing model fairness is a significant yet unresolved requirement for the safe implementation of UAD models. Towards this end, the sAUROC presented here is a relevant contribution, as it facilitates the quantification of performance bias in UAD models. We emphasise that sAUROC is also relevant for supervised classification models where disparities between subgroups also manifest in TPR/FPR shifts.^7^^,^^17^ Considering the severe implications that over-diagnosis can have on both society and individual patients, we believe the quantification of existing bias mechanisms in this work presents a vital step towards a fairer future in healthcare.

## Contributors

F.M. conducted the experiments. F.M., S.B., M.K., and B.W. conceptualised the study, performed literature search, and validated and interpreted the experimental results. F.M. and M.K. verified the underlying data. G.K., R.M., and D.R. acquired funding, provided resources, and supervised the study with regular feedback. All authors contributed to writing and critical revision of the manuscript and have read and approved the final version of the manuscript.

## Data sharing statement

All data used in this work is publicly available. The CXR14 dataset, together with the patient demographic information, can be downloaded from https://nihcc.app.box.com/v/ChestXray-NIHCC/folder/36938765345. For the CheXpert dataset, analogous information is available from https://stanfordmlgroup.github.io/competitions/chexpert/. The MIMIC-CXR dataset can be downloaded from https://physionet.org/content/mimic-cxr-jpg/2.0.0/with the corresponding patient demographic information available from https://physionet.org/content/mimiciv/2.2/. All information to reproduce the exact results in this paper is available under an open-source Apache 2.0 license in our dedicated GitHub repository https://github.com/FeliMe/unsupervised_fairness.

## Declaration of interests

B.W. has received speaker honoraria from Novartis, Bayer and Philips. He holds a patent related to Apogenix APG101 for glioblastoma treatment and is a stockholder of the company “Need”.

## Footnotes

k Similar to “scaling laws” for transformers in natural image processing tasks.^12^

l Categories like diverse/intersex/non-binary were missing in the data.
