# Supplementary material for: (Predictable) performance bias in unsupervised anomaly detection
Source: eBioMedicine. 2024 Feb 9;101:105002. doi: 10.1016/j.ebiom.2024.105002 (PMC10873649; doi:10.1016/j.ebiom.2024.105002)
Supplement: Supplementary Figures [file mmc1.docx]

# Supplementary figures

Figure S1. **Experimental results for RD on the MIMIC-CXR, CXR14, and CheXpert datasets trained under different sex, age, or race imbalance ratios.** Each box represents the results over ten runs with different random seeds. Regression lines along the different imbalance ratios are additionally plotted. The correlation of the performance of a subgroup with its representation in the training data was strong for the protected attributes sex and age (Pearson correlation coefficient 0·931 and 0·931) and notable for race (0·66). The mean absolute errors when linearly interpolating between the extreme dataset compositions was 0·0089±0·0039 for sex, 0·0083±0·0039 for age, and 0·0042±0·0014 for race. With balanced training data (50% from each subgroup), one subgroup almost always performed significantly better (Welch’s t-test, p < 0·01, N=10). Only the difference between the male and female subgroups in MIMIC-CXR was not significant.
