# Supplementary material for: (Predictable) performance bias in unsupervised anomaly detection
Source: eBioMedicine. 2024 Feb 9;101:105002. doi: 10.1016/j.ebiom.2024.105002 (PMC10873649; doi:10.1016/j.ebiom.2024.105002)
Supplement: Supplementary Tables [file mmc2.docx]

# Supplementary tables

Table S1. Patient demographics (gender, age, and race) of the MIMIC-CXR, CXR14, and CheXpert datasets after inclusion criteria.

|  | MIMIC-CXR | CXR14 | CheXpert |
| --- | --- | --- | --- |
| Total images | 377 110 | 112 120 | 223 648 |
| Frontal view | 230 721 | 112 120 | 191 229 |
| W/o support devices | 157 371 | 112 120 | 77 602 |
| W/o uncertain labels | 151 450 | 112 120 | 76 635 |
| Normal images | 65 033 | 60 361 | 8920 |
| Female | 33 084 | 26 439 | 3768 |
| Male | 31 949 | 33 922 | 5151 |
| Young | 9574 | 13 169 | 1706 |
| Old | 24 731 | 11 971 | 2711 |
| Black | 10 834 | N/A | 646 |
| White | 29 329 | N/A | 4565 |
| Abnormal images | 86 417 | 51 759 | 66 715 |
| Female | 40 417 | 22 341 | 28 755 |
| Male | 46 000 | 29 418 | 47 959 |
| Young | 3766 | 9703 | 4420 |
| Old | 54 319 | 12 692 | 40 091 |
| Black | 12 076 | N/A | 3820 |
| White | 48 785 | N/A | 38 208 |

Table S2. Patient demographics (sex, age, and race) of the MIMIC-CXR, CXR14, and CheXpert datasets after inclusion criteria.

|  | **train / val /test** | | |
| --- | --- | --- | --- |
|  | MIMIC-CXR | CXR14 | CheXpert |
| Sex | 28 862 / 2000 / 2000 | 21 268 / 2000 / 2000 | 3204 / 1000 / 1000 |
| Age | 7536 / 2000 / 2000 | 7586 / 2000 / 2000 | 1136 / 1000 / 1000 |
| Race | 7966 / 2000 / 2000 | N/A | N/A |

Table S3. Number of samples in the test sets of the intersectional experiments.

| Subgroup set | # samples |
| --- | --- |
| Train | 35 744 |
| Test female | 1000 |
| Test female old | 500 |
| Test female young | 500 |
| Test female black | 500 |
| Test female white | 500 |
| Test male | 1000 |
| Test male old | 500 |
| Test male young | 500 |
| Test male black | 500 |
| Test male white | 500 |
| Test young | 1000 |
| Test young | 500 |
| Test young | 500 |
| Test old | 1000 |
| Test old | 500 |
| Test old | 500 |
| Test black | 1000 |
| Test white | 1000 |

Table S4. Hyperparameters for the structural Feature-Autoencoder (FAE) used in all experiments.

| Name | Value |
| --- | --- |
| Learning rate | 0·0002 |
| Weight decay | 0·0 |
| # channels per layer | 100, 150, 200, 250, 300, 250, 200, 150, 100 |
| Dropout rate | 0·1 |
| Feature extractor | ResNet18 |
| Extractor layers | Layer0, layer1, layer2 |
| Keep feature fraction | 1·0 |

Table S5. Hyperparameters for the Reverse Distillation (RD) model used in all experiments.

| Name | Value |
| --- | --- |
| Learning rate | 0·0002 |
| Weight decay | 0·0 |
| Backbone | ResNet18 |

Table S6. Results of FAE for female and male patients with varying proportions of male subjects during training. sAUROC means and standard deviations over 10 runs with different seeds.

|  | **MIMIC-CXR** | | **CXR14** | | **CheXpert** | |
| --- | --- | --- | --- | --- | --- | --- |
| Male % | male | female | male | female | male | female |
| 0·0 | 0·691±0·003 | 0·738±0·003 | 0·668±0·003 | 0·644±0·002 | 0·721±0·005 | 0·749±0·003 |
| 0·1 | 0·709±0·003 | 0·725±0·004 | 0·686±0·002 | 0·630±0·002 | 0·737±0·005 | 0·738±0·004 |
| 0·2 | 0·715±0·003 | 0·713±0·005 | 0·693±0·002 | 0·619±0·003 | 0·741±0·003 | 0·730±0·004 |
| 0·3 | 0·721±0·002 | 0·698±0·014 | 0·701±0·002 | 0·613±0·003 | 0·748±0·003 | 0·727±0·003 |
| 0·4 | 0·726±0·002 | 0·673±0·010 | 0·705±0·001 | 0·605±0·002 | 0·751±0·003 | 0·720±0·003 |
| 0·5 | 0·732±0·001 | 0·657±0·002 | 0·709±0·002 | 0·598±0·003 | 0·755±0·002 | 0·715±0·002 |
| 0·6 | 0·736±0·001 | 0·650±0·002 | 0·714±0·002 | 0·593±0·002 | 0·757±0·003 | 0·709±0·004 |
| 0·7 | 0·740±0·002 | 0·642±0·003 | 0·718±0·002 | 0·583±0·002 | 0·759±0·003 | 0·702±0·004 |
| 0·8 | 0·744±0·001 | 0·633±0·003 | 0·724±0·001 | 0·576±0·003 | 0·766±0·003 | 0·695±0·005 |
| 0·9 | 0·750±0·001 | 0·619±0·001 | 0·729±0·002 | 0·564±0·002 | 0·772±0·002 | 0·680±0·003 |
| 1·0 | 0·757±0·002 | 0·596±0·003 | 0·737±0·001 | 0·551±0·002 | 0·780±0·002 | 0·664±0·003 |

Table S7. Results of FAE for young and old patients with varying proportions of old subjects during training. sAUROC means and standard deviations over 10 runs with different seeds.

|  | **MIMIC-CXR** | | **CXR14** | | **CheXpert** | |
| --- | --- | --- | --- | --- | --- | --- |
| Old % | old | young | old | young | old | young |
| 0·0 | 0·597±0·002 | 0·781±0·004 | 0·622±0·002 | 0·673±0·006 | 0·639±0·005 | 0·742±0·005 |
| 0·1 | 0·608±0·002 | 0·764±0·005 | 0·628±0·003 | 0·656±0·007 | 0·640±0·003 | 0·727±0·005 |
| 0·2 | 0·615±0·002 | 0·757±0·003 | 0·634±0·002 | 0·648±0·004 | 0·647±0·004 | 0·719±0·004 |
| 0·3 | 0·618±0·002 | 0·752±0·007 | 0·635±0·004 | 0·637±0·006 | 0·654±0·003 | 0·712±0·003 |
| 0·4 | 0·622±0·003 | 0·747±0·004 | 0·639±0·004 | 0·627±0·002 | 0·660±0·003 | 0·707±0·004 |
| 0·5 | 0·624±0·002 | 0·742±0·006 | 0·644±0·003 | 0·616±0·006 | 0·662±0·004 | 0·702±0·001 |
| 0·6 | 0·628±0·002 | 0·740±0·006 | 0·648±0·002 | 0·610±0·003 | 0·671±0·003 | 0·694±0·004 |
| 0·7 | 0·631±0·002 | 0·735±0·005 | 0·651±0·002 | 0·605±0·003 | 0·671±0·003 | 0·690±0·003 |
| 0·8 | 0·635±0·003 | 0·727±0·006 | 0·653±0·003 | 0·597±0·004 | 0·675±0·004 | 0·672±0·006 |
| 0·9 | 0·642±0·004 | 0·719±0·004 | 0·657±0·002 | 0·588±0·003 | 0·679±0·003 | 0·661±0·005 |
| 1·0 | 0·655±0·003 | 0·692±0·003 | 0·664±0·002 | 0·572±0·004 | 0·688±0·004 | 0·646±0·004 |

Table S8. Results of FAE for black and white patients with varying proportions of white subjects during training. sAUROC means and standard deviations over 10 runs with different seeds.

|  | **MIMIC-CXR** | |
| --- | --- | --- |
| White % | white | black |
| 0·0 | 0·682±0·002 | 0·725±0·003 |
| 0·1 | 0·683±0·003 | 0·720±0·005 |
| 0·2 | 0·685±0·002 | 0·716±0·004 |
| 0·3 | 0·686±0·003 | 0·706±0·010 |
| 0·4 | 0·685±0·003 | 0·695±0·011 |
| 0·5 | 0·684±0·003 | 0·682±0·005 |
| 0·6 | 0·686±0·004 | 0·686±0·010 |
| 0·7 | 0·684±0·003 | 0·673±0·005 |
| 0·8 | 0·685±0·002 | 0·670±0·005 |
| 0·9 | 0·686±0·002 | 0·665±0·002 |
| 1·0 | 0·686±0·001 | 0·658±0·002 |

Table S9. Pearson correlation coefficients of different representations of a particular subgroup in the training data for every subgroup on all datasets for the FAE model. Mean and standard deviation over 10 runs with different random seeds.

|  | MIMIC-CXR | CXR14 | CheXpert |
| --- | --- | --- | --- |
| Male | 0·977±0·005 | 0·976±0·006 | 0·964±0·011 |
| Female | 0·986±0·005 | 0·993±0·003 | 0·977±0·005 |
| Young | 0·950±0·020 | 0·986±0·005 | 0·976±0·008 |
| Old | 0·969±0·008 | 0·975±0·015 | 0·970±0·008 |
| Black | 0·966±0·010 | - | - |
| White | 0·398±0·241 | - | - |

Table S10. Results of RD for female and male patients with varying proportions of male subjects during training. sAUROC means and standard deviations over 10 runs with different seeds.

|  | **MIMIC-CXR** | | **CXR14** | | **CheXpert** | |
| --- | --- | --- | --- | --- | --- | --- |
| Male % | male | female | male | female | male | female |
| 0·0 | 0·638±0·004 | 0·694±0·004 | 0·609±0·004 | 0·604±0·003 | 0·651±0·011 | 0·679±0·005 |
| 0·1 | 0·651±0·004 | 0·687±0·004 | 0·617±0·004 | 0·598±0·004 | 0·655±0·010 | 0·668±0·009 |
| 0·2 | 0·657±0·004 | 0·684±0·004 | 0·622±0·004 | 0·593±0·003 | 0·669±0·007 | 0·663±0·009 |
| 0·3 | 0·661±0·003 | 0·682±0·004 | 0·629±0·005 | 0·586±0·004 | 0·669±0·012 | 0·658±0·009 |
| 0·4 | 0·667±0·002 | 0·680±0·004 | 0·630±0·004 | 0·580±0·004 | 0·674±0·008 | 0·663±0·011 |
| 0·5 | 0·671±0·003 | 0·675±0·004 | 0·638±0·003 | 0·577±0·004 | 0·680±0·011 | 0·644±0·010 |
| 0·6 | 0·678±0·004 | 0·674±0·004 | 0·641±0·003 | 0·575±0·004 | 0·692±0·010 | 0·643±0·011 |
| 0·7 | 0·680±0·001 | 0·669±0·004 | 0·647±0·004 | 0·568±0·005 | 0·695±0·010 | 0·637±0·010 |
| 0·8 | 0·684±0·003 | 0·663±0·003 | 0·651±0·005 | 0·557±0·004 | 0·699±0·010 | 0·626±0·008 |
| 0·9 | 0·690±0·003 | 0·657±0·003 | 0·660±0·002 | 0·550±0·004 | 0·711±0·011 | 0·626±0·009 |
| 1·0 | 0·699±0·006 | 0·640±0·003 | 0·671±0·004 | 0·524±0·004 | 0·726±0·007 | 0·605±0·010 |

Table S11. Results of RD for young and old patients with varying proportions of old subjects during training. sAUROC means and standard deviations over 10 runs with different seeds.

|  | **MIMIC-CXR** | | **CXR14** | | **CheXpert** | |
| --- | --- | --- | --- | --- | --- | --- |
| Old % | old | young | old | young | old | young |
| 0·0 | 0·545±0·004 | 0·736±0·003 | 0·577±0·004 | 0·623±0·003 | 0·570±0·010 | 0·673±0·009 |
| 0·1 | 0·553±0·004 | 0·727±0·003 | 0·587±0·005 | 0·616±0·005 | 0·586±0·007 | 0·656±0·010 |
| 0·2 | 0·560±0·004 | 0·723±0·004 | 0·594±0·006 | 0·607±0·003 | 0·587±0·011 | 0·640±0·008 |
| 0·3 | 0·559±0·004 | 0·716±0·004 | 0·601±0·006 | 0·605±0·005 | 0·593±0·011 | 0·638±0·010 |
| 0·4 | 0·562±0·004 | 0·708±0·004 | 0·602±0·005 | 0·602±0·006 | 0·591±0·009 | 0·634±0·009 |
| 0·5 | 0·566±0·004 | 0·705±0·003 | 0·606±0·005 | 0·596±0·004 | 0·596±0·010 | 0·623±0·012 |
| 0·6 | 0·570±0·005 | 0·698±0·003 | 0·610±0·004 | 0·593±0·004 | 0·598±0·012 | 0·617±0·012 |
| 0·7 | 0·571±0·005 | 0·692±0·006 | 0·614±0·004 | 0·589±0·004 | 0·597±0·008 | 0·609±0·009 |
| 0·8 | 0·574±0·005 | 0·682±0·005 | 0·617±0·005 | 0·582±0·005 | 0·597±0·015 | 0·596±0·011 |
| 0·9 | 0·576±0·006 | 0·674±0·005 | 0·620±0·003 | 0·577±0·005 | 0·602±0·012 | 0·584±0·009 |
| 1·0 | 0·590±0·006 | 0·653±0·006 | 0·626±0·004 | 0·564±0·005 | 0·614±0·007 | 0·564±0·009 |

Table S12. Results of RD for black and white patients with varying proportions of white subjects during training. sAUROC means and standard deviations over 10 runs with different seeds.

|  | **MIMIC-CXR** | |
| --- | --- | --- |
| White % | white | black |
| 0·0 | 0·638±0·004 | 0·692±0·003 |
| 0·1 | 0·638±0·005 | 0·692±0·005 |
| 0·2 | 0·641±0·005 | 0·690±0·003 |
| 0·3 | 0·641±0·004 | 0·685±0·003 |
| 0·4 | 0·643±0·003 | 0·685±0·004 |
| 0·5 | 0·646±0·003 | 0·686±0·005 |
| 0·6 | 0·644±0·004 | 0·681±0·003 |
| 0·7 | 0·646±0·004 | 0·681±0·003 |
| 0·8 | 0·643±0·004 | 0·676±0·003 |
| 0·9 | 0·644±0·004 | 0·678±0·005 |
| 1·0 | 0·647±0·003 | 0·673±0·004 |

Table S13. Pearson correlation coefficients of different representations of a particular subgroup in the training data for every subgroup on all datasets for the RD model. Mean and standard deviation over 10 runs with different random seeds.

|  | MIMIC-CXR | CXR14 | CheXpert |
| --- | --- | --- | --- |
| Male | 0·973±0·010 | 0·972±0·012 | 0·915±0·042 |
| Female | 0·928±0·030 | 0·948±0·014 | 0·908±0·050 |
| Young | 0·973±0·010 | 0·960±0·015 | 0·948±0·022 |
| Old | 0·924±0·032 | 0·952±0·025 | 0·648±0·163 |
| Black | 0·848±0·072 | - | - |
| White | 0·537±0·228 | - | - |
